# Anthropometric and Metabolic Risk Factors for ESRD Are Disease-Specific: Results from a Large Population-Based Cohort Study in Austria

**DOI:** 10.1371/journal.pone.0161376

**Published:** 2016-08-18

**Authors:** Emanuel Zitt, Constanze Pscheidt, Hans Concin, Reinhard Kramar, Karl Lhotta, Gabriele Nagel

**Affiliations:** 1 Department of Nephrology and Dialysis, Academic Teaching Hospital Feldkirch, Feldkirch, Austria; 2 Vorarlberg Institute for Vascular Investigation and Treatment (VIVIT), Academic Teaching Hospital Feldkirch, Feldkirch, Austria; 3 Agency for Preventive and Social Medicine, Bregenz, Austria; 4 Institute of Epidemiology and Medical Biometry, Ulm University, Ulm, Germany; 5 Austrian Dialysis and Transplant Registry, Rohr im Kremstal, Austria; Graduate School of Medicine, University of the Ryukyus, JAPAN

## Abstract

**Background:**

Anthropometric and metabolic risk factors for all-cause end-stage renal disease (ESRD) may vary in their impact depending on the specific primary renal disease.

**Methods:**

In this Austrian population-based prospective cohort study (n = 185,341; 53.9% women) the following data were collected between 1985 and 2005: age, sex, body mass index (BMI), fasting blood glucose (FBG) from 1988, blood pressure, total cholesterol (TC), triglycerides (TG), gamma-glutamyl transferase (GGT) and smoking status. These data were merged with the Austrian Dialysis and Transplant Registry to identify ESRD patients. Cox proportional hazards models were applied to calculate hazard ratios (HR) for all-cause ESRD as well as for cause-specific ESRD due to the following primary renal diseases: autosomal dominant polycystic kidney disease (ADPKD), vascular nephropathy (VN), diabetic nephropathy (DN) and other diseases (OD).

**Results:**

During a mean follow-up of 17.5 years 403 participants developed ESRD (ADPKD 36, VN 97, DN 86, and OD 184). All parameters except TG and GGT were significantly associated with all-cause ESRD risk. Particular cause-specific ESRD risk factor patterns were found: for ADPKD increased risk from hypertension (HR 11.55); for VN from smoking (HR 1.81), hypertension (HR 2.37), TG (≥5.70 vs. <1.17 mmol/L: HR 9.27); for DN from smoking (HR 1.77), BMI (≥30 vs. 18.5–24.9 kg/m^2^: HR 7.55), FBG (≥6.94 vs. <5.55 mmol/L: HR 7.67), hypertension (HR 1.08), TG (≥5.70 vs. <1.17 mmol/L: HR 2.02), GGT (HR 2.14); and for OD from hypertension (HR 2.29), TG (≥5.70 vs. <1.17 mmol/L: HR 6.99) and TC (≥6.22 vs. <5.18 mmol/L: HR 1.56).

**Conclusions:**

Particular anthropometric and metabolic ESRD risk factors differ in importance depending on the primary renal disease. This needs to be considered for future preventive and therapeutic strategies addressing cause-specific ESRD.

## Introduction

In recent years, efforts to promote the early identification and treatment of patients at risk for and suffering from chronic kidney disease have stabilized the number of incident end-stage renal disease (ESRD) patients in most industrialized countries. Nevertheless, the number of prevalent patients on renal replacement therapy (RRT) is still increasing [1, 2]. For the implementation of effective preventive measures, the identification of relevant risk factors for ESRD is paramount. Several studies have addressed these long-term predictors and risk factors for ESRD, such as body mass index, blood pressure, smoking and metabolic factors like hyperglycemia, hyperlipidemia or hyperuricemia [3–9]. These studies analysed all-cause ESRD as a single entity and did not differentiate between the underlying renal diseases. Risk factors and the effectiveness of therapeutic interventions may, however, differ for cause-specific ESRD.

The aim of the present study in a large population-based cohort was to investigate whether accepted anthropometric and metabolic risk factors for all-cause ESRD vary in their impact on cause-specific ESRD.

## Methods

### Study population

The study population has been previously described in detail [10]. The Vorarlberg Health Monitoring & Prevention Programme (VHM&PP) is a population-based risk factor surveillance program in Vorarlberg [11, 12]. All adults in the westernmost Austrian state (approx. 400.000 inhabitants) were invited to participate. Enrolment was voluntary and costs for one examination per year are covered by the participant’s compulsory health insurance. Between January 1, 1985 and June 30, 2005 185,341 inhabitants had at least one health examination (HE). The screening examinations were conducted in the practices of local physicians according to a standard protocol. Height, body weight, systolic blood pressure (BPsys) and diastolic blood pressure (BPdia) were determined. Blood glucose (BG), total cholesterol (TC), triglycerides (TG) and gamma-glutamyl transferase (GGT) were measured (since January 1, 1988) in an overnight fasting blood sample. Information on smoking status was collected in a standardized interview.

Data from the VHM&PP cohort were then merged with the data in the Austrian Dialysis and Transplant Registry (OEDTR). This registry kept by the Austrian Society of Nephrology, established in 1964, collects data on all patients entering chronic renal replacement therapy in Austria [13]. Between January 1, 1985 and December 31, 2009 813 patients from Vorarlberg were included in the registry, 403 of whom had also participated in VHM&PP.

The study was performed according to the Declaration of Helsinki of the World Medical Association. Ethics approval for the study was obtained from the Ethics Committee of the State of Vorarlberg. All patients registered in the OEDTR signed a declaration of consent to permit their data to be transferred to the registry.

### Exposure variables

To provide information on clinically relevant cut-points, we calculated the models including categorical variables: BMI was calculated as weight in kilograms divided by squared height in meters (kg/m^2^) and categorised [14]: <18.5, ≥18.5 - <25, ≥25 - <30- and ≥30. Blood glucose levels (mmol/L) were categorised: <5.55, ≥5.55 - <6.94 and ≥6.94 [15]. Categories for triglycerides (mmol/L) were: <1.17, ≥1.17 - <2.28, ≥2.28 - <5.70 and ≥5.70. Total cholesterol (mmol/L) was categorised: <5.18, ≥5.18 - <6.22 and ≥6.22 [16]. For gamma-GT sex-specific cutoffs were used. For men, elevated gamma-GT values were defined as >60 U/L, for women >35 U/L. Obesity was defined as body mass index ≥30 kg/m², and hypertension as systolic blood pressure ≥140 mmHg or diastolic blood pressure ≥90 mmHg. Due to skewness GGT and TG values were logarithmized.

### Outcome variables

Outcome was defined as ESRD requiring chronic RRT with dialysis or transplantation.

The primary underlying renal disease for cause-specific ESRD was retrieved from the ERA-EDTA Primary Renal Diagnosis Codes provided by the OEDTR as follows: autosomal dominant polycystic kidney disease (ADPKD) code 41; vascular/hypertensive nephropathy (VN) codes 70 (renal vascular disease, type unspecified), 71 (renal vascular disease due to malignant hypertension) and 72 (renal vascular disease due to hypertension); type 2 diabetes with nephropathy (DN) code 81 (diabetic glomerulosclerosis or diabetic nephropathy type 2). The remainder including chronic glomerulonephritis and interstitial nephropathies or other hereditary nephropathies were classified as other diseases (OD).

### Statistical Analysis

We calculated the overall and cause-specific event rates by categories of the independent variables. Follow-up began after the baseline examination and ended at ESRD diagnosis, death or the censoring date (December 31, 2009), whichever occurred first. Cox proportional hazards models were fitted to calculate hazard ratios (HR) with a 95% confidence interval (CI) for all-cause and cause-specific ESRD related to metabolic risk factors. We estimated HRs for categorical variables defined by clinically meaningful cut-off points and for continuous exposure variables. In addition, we estimated the nonlinear association between metabolic factors and ESRD using cubic restricted splines with knots at the 5%, 35%, 65% and 95% percentiles of the exposure variables. All models were adjusted for age, sex and smoking status and were additionally adjusted for all risk factors simultaneously. The possibility of effect modification was examined by stratifying by BMI, and interactions were tested for continuous exposure variables, but not found to influence the results. P values < .05 were considered statistically significant. Nonlinear associations were modelled using R (R Foundation for Statistical Computing, Vienna, Austria), Version 3.0.1. All other calculations were performed using the statistical analysis software SAS, Release 9.3 (SAS Institute, Cary, NC, USA).

## Results

Of the study population of 185,341 persons 53.9% were women and mean age at baseline was 41.6±15.3 years. During a mean follow-up of 17.5±6.1 years 22,665 persons (49% women) died and 403 (39.2% women) of these individuals developed ESRD and commenced RRT. Mean age at ESRD was 63.4±12.7 years and mean time between baseline and ESRD onset was 11.8±6.3 years. The specific ESRD cause in these 403 patients was: ADPKD in 36 patients (9%), VN in 97 patients (24%), DN in 86 patients (21%) and OD) in 184 patients (46%). Table 1 shows the characteristics of the study population by cause-specific ESRD categories. Patients who developed ESRD during the follow-up were ten years older at baseline investigation. Whereas women were predominantly present (53.9%) in the whole study population, the majority of ESRD patients were men (60.8%), except in the ADPKD group which showed almost equal numbers of women and men. Smokers were more prevalent in the ESRD group (35.7% vs 28.8%, P < .001). Moreover, the proportion of obese participants was higher in the patient group who developed ESRD (21.3% vs 10.7%, P<0.001) due to the high obesity rate in the diabetic nephropathy group, and they twice as often suffered from hypertension (74.9% vs 36.4%, P< .001). The event rates for all-cause ESRD per 100.000 patient years were higher for men, smokers, and participants with increasing BMI, glucose, TG, TC and GGT levels (Table 2). Cause-specific ESRD event rates throughout the different risk factor categories are presented in Table 2. Men had highest event rates for VN. DN was characterized by high event rates in obese patients and patients with high glucose levels and increased gamma-GT. For high levels of TG high event rates for VN, DN and OD were observed.

**Table 1:** Baseline characteristics of study population.

	Entire cohort	No ESRD	All-cause ESRD	Cause-specific ESRD										
				ADPKD	VN	DN	Other diseases							
	**N**	**Mean (SD)**	**N**	**Mean (SD)**	**N**	**Mean (SD)**	**N**	**Mean (SD)**	**N**	**Mean (SD)**	**N**	**Mean (SD)**	**N**	**Mean (SD)**
**Age at baseline [years]**	185,341	41.6 (15.3)	184,938	41.6 (15.3)	403	51.6 (12.1)	36	46.7 (10.6)	97	58.0 (10.4)	86	52.6 (9.8)	184	48.8 (12.9)
**Follow-up [years]**	185,341	17.5 (6.1)	184,938	17.5 (6.1)	403	20.0 (4.8)	36	21.4 (4.3)	97	20.0 (4.9)	86	20.5 (4.5)	184	19.5 (4.9)
**Age at ESRD [years]**	403	63.4 (12.7)	-	-	403	63.4 (12.7)	36	57.9 (10.4)	97	71.3 (10.7)	86	66.1 (8.7)	184	59.1 (13.4)
**Time interval baseline—ESRD [years]**	403	11.8 (6.3)	-	-	403	11.8 (6.3)	36	11.3 (5.8)	97	13.3 (6.2)	86	13.5 (6.2)	184	10.3 (6.1)
	**N**	**%**	**N**	**%**	**N**	**%**	**N**	**%**	**N**	**%**	**N**	**%**	**N**	**%**
**Sex**														
Female	99,881	53.9	99,723	53.9	158	39.2	19	52.8	28	28.9	37	43.0	74	40.2
Male	85,460	46.1	85,215	46.1	245	60.8	17	47.2	69	71.1	49	57.0	110	59.8
**Smoking status**														
Non-smoker	131,969	71.2	131,710	71.2	259	64.3	24	66.7	57	58.8	49	57.0	129	70.1
Smoker	53,372	28.8	53,228	28.8	144	35.7	12	33.3	40	41.2	37	43.0	55	29.9
**Obesity** *														
No	165,321	89.2	165,004	89.3	317	78.7	33	91.7	83	85.6	49	57.0	152	82.6
Yes	19,969	10.8	19,883	10.7	86	21.3	3	8.3	14	14.4	37	43.0	32	17.4
**Hypertension** *														
No	117,658	63.5	117,557	63.6	101	25.1	5	13.9	22	22.7	16	18.6	58	31.7
Yes	67,506	36.5	67,205	36.4	301	74.9	31	86.1	75	77.3	70	81.4	125	68.3

SD, standard deviation; ESRD, end-stage renal disease; ADPKD, autosomal dominant polycystic kidney disease; VN, vascular nephropathy; DN, diabetic nephropathy.

*data does not sum up due to missing values

**Table 2:** Event rates for all-cause and cause-specific ESRD.

				All-cause ESRD	Cause-specific ESRD				
Risk factor					ADPKD	VN	DN	Other disease	
	Categories	Person years	# Deaths	N	Event rate*	Event rate*			
**Sex**	Women	1,684,358	11,079	158	9.38	1.13	1.66	2.20	4.39
	Men	1,357,254	11,383	245	18.05	1.25	5.08	3.61	8.10
**Age [years]**	< 30	860,027	461	17	1.98	0.23	0.12	-	1.63
	30–39	744,028	1,048	58	7.80	1.08	0.81	1.21	4.70
	40–49	616,195	2,384	110	17.85	2.11	2.60	5.03	8.11
	50–59	460,092	4,564	103	22.39	1.74	5.87	4.78	10.00
	60–69	255,646	6,765	94	36.77	1.96	14.47	8.61	11.73
	≥70	105,625	7,240	21	19.88	-	9.47	1.89	8.52
**Smoking status**	Non-smokers	2,149,272	16,166	259	12.05	1.12	2.65	2.28	6.00
	Smokers	892,341	6,296	144	16.14	1.34	4.48	4.15	6.16
**BMI [kg/m**^**2**^**]**	<18.50	96,673	485	1	1.03	-	-	-	1.03
	18.50 - < 25	1,700,328	9,103	133	7.82	1.00	2.00	0.65	4.18
	25 - < 30	940,866	9,143	183	19.45	1.70	5.21	4.04	8.50
	≥ 30	302,958	3,721	86	28.39	0.99	4.62	12.21	10.56
**Glucose [mmol/L]**	< 5.55	1,789,414	9,034	154	8.61	0.78	2.29	0.45	5.09
	5.55 - < 6.94	575,810	4,510	74	12.85	0.69	3.30	1.56	7.29
	≥ 6.94	654,077	8,245	158	24.16	2.45	5.50	9.02	7.19
**Hypertension**	No	1,951,682	7,004	101	5.18	0.26	1.13	0.82	2.97
	Yes	1,086,851	15,434	301	27.69	2.85	6.90	6.44	11.50
**Triglycerides [mmol/L]**	<1.17	1,440,477	6,803	74	5.14	0.56	1.18	0.62	2.78
	1.17 - < 2.28	1,133,043	10,301	179	15.80	2.03	5.53	2.74	7.50
	2.28 - < 5.70	379,215	4,513	120	31.64	1.32	7.91	9.49	12.92
	≥5.70	31,541	400	24	76.09	-	25.36	25.36	25.36
**Cholesterol [mmol/L]**	<5.18	1,174,284	4,456	71	6.05	1.11	1.19	0.60	3.15
	5.18 - < 6.22	985,051	7,215	123	12.49	1.22	3.15	2.94	5.18
	≥ 6.22	829,151	10,398	203	24.48	1.33	6.03	5.79	11.34
**Gamma-GT [U/L]**	normal	2,604,467	16,474	297	11.40	1.19	2.92	1.69	5.61
	men: > 60 U/L, women: >35 U/L	380,064	5,538	100	26.31	1.32	5.00	10.52	9.47

*per 100,000 person years;

– no patients in this subgroup;

ADPKD, autosomal dominant polycystic kidney disease; VN, vascular nephropathy; DN, diabetic nephropathy; BMI, body mass index.

Table 3 shows the hazard ratios (HR) for smoking status, anthropometric and metabolic factors for all-cause ESRD and the different cause-specific ESRD categories in 380 patients with a complete data set for all exposure variables.

**Table 3:** Hazard ratios (HR) for all-cause and cause-specific ESRD for discrete variables.

			Cause-specific ESRD			
Risk factor	Categories	All-cause ESRD	ADPKD	VN	DN	Other disease
		HR (95% CI)	HR (95% CI)	HR (95% CI)	HR (95% CI)	HR (95% CI)
N = 181,139*		380	34	94	75	177
**Sex**	Women	1.00	1.00	1.00	1.00	1.00
	Men	1.52 (1.23–1.93)	0.90 (0.44–1.83)	2.90 (1.79–4.73)	0.88 (0.54–1.47)	1.56 (1.13–2.15)
**Smoking status**	Non-smokers	1.00	1.00	1.00	1.00	1.00
	Smokers	1.32 (1.06–1.65)	1.41 (0.68–2.94)	1.81 (1.17–2.81)	1.77 (1.07–2.91)	0.98 (0.70–1.36)
**BMI [kg/m**^**2**^**]**	<18.50	0.33 (0.05–2.36)	–	–	–	0.51 (0.07–3.68)
	18.50 - < 25	1.00	1.00	1.00	1.00	1.00
	25 - < 30	1.16 (0.91–1.48)	0.79 (0.37–1.67)	1.11 (0.70–1.77)	3.26 (1.41–7.53)	1.08 (0.77–1.53)
	≥30	1.43 (1.06–1.92)	0.37 (0.10–1.23)	0.89 (0.46–1.71)	7.55 (3.23–17.61)	1.19 (0.76–1.87)
**Glucose [mmol/L]**	< 5.55	1.00	1.00	1.00	1.00	1.00
	5.55 - < 6.94	1.12 (0.84–1.48)	0.82 (0.27–2.51)	0.97 (0.56–1.68)	2.29 (0.88–5.96)	1.17 (0.81–1.69)
	≥ 6.94	1.41 (1.12–1.78)	2.51 (1.18–5.34)	0.94 (0.59–1.51)	7.67 (3.60–16.30)	0.84 (0.58–1.21)
**Hypertension**	No	1.00	1.00	1.00	1.00	1.00
	Yes	2.61 (2.02–3.36)	11.55 (4.22–31.60)	2.37 (1.41–3.97)	2.23 (1.20–4.14)	2.29 (1.61–3.25)
**Triglycerides [mmol/L]**	<1.17	1.00	1.00	1.00	1.00	1.00
	1.17 - < 2.28	1.88 (1.40–2.51)	2.89 (1.22–6.69)	1.70 (0.94–3.09)	2.01 (0.89–4.78)	1.82 (1.22–2.72)
	2.28 - < 5.70	2.65 (1.90–3.69)	1.92 (0.57–6.49)	2.86 (1.48–5.53)	3.73 (1.57–8.93)	2.36 (1.47–3.81)
	≥5.70	5.58 (3.35–9.32)	–	9.27 (3.68–23.36)	6.99 (2.26–21.63)	4.36 (1.92–9.87)
**Cholesterol [mmol/L]**	<5.18	1.00	1.00	1.00	1.00	1.00
	5.18 - < 6.22	1.13 (0.83–1.53)	0.66 (0.29–1.48)	1.24 (0.64–2.40)	2.39 (0.98–5.80)	1.06 (0.68–1.65)
	≥6.22	1.22 (0.90–1.65)	0.40 (0.16–1.02)	1.26 (0.66–2.39)	1.77 (0.73–4.30)	1.56 (1.02–2.41)
**Gamma-GT [U/L]**	Normal	1.00	1.00	1.00	1.00	1.00
	Men: > 60 U/L, Women: >35 U/L	1.09 (0.85–1.39)	0.81 (0.31–2.15)	0.82 (0.48–1. 38)	2.14 (1.33–3.43)	0.92 (0.62–1.35)

All models are adjusted for all risk factors and age (years);

*with full set of covariates;

– no patients in this subgroup;

ADPKD, autosomal dominant polycystic kidney disease; VN, vascular nephropathy; DN, diabetic nephropathy; CI, confidence interval; BMI, body mass index.

Male sex, smoking, BMI over 30 kg/m^2^, FBG levels over 6.94 mmol/L, hypertension, and TG over 1.17 mmol/L increased the risk for all-cause ESRD.

Detailed analysis revealed marked cause-specific ESRD risk patterns: Hypertension significantly increased the risk for ESRD due to ADPKD (HR 11.55, 95% CI 4.22–31.6), as well as increased FBG levels (≥6.94 mmol/L vs. reference (Ref.): HR 2.51; 1.18–5.34) and TG levels (1.17- <2.28 mmol/L vs. Ref.: HR 2.89, 1.22–6.69). Risk factors for ESRD caused by VN were male sex (HR 2.90, 1.79–4.73), smoking (HR 1.81, 1.17–2.81) and TG (2.28 - <5.70 mmol/L vs. Ref.: HR 2.86, 1.48–5.53; and ≥5.70 mmol/L vs. Ref.: HR 9.27, 3.68–23.36). For ESRD caused by DN risk increased with smoking (HR 1.77, 1.07–2.91), elevated BMI (25 - <30 kg/m^2^ vs. Ref.: HR 3.26, 1.41–7.53; and ≥30 vs. Ref.: HR 7.55, 3.23–17.61), increased FBG (≥6.94 mmol/L vs. Ref.: HR 7.67, 3.60–16.30) and TG levels (2.28 - <5.70mmol/L vs. Ref.: HR 3.73, 1.57–8.93; and ≥5.70 mmol/L vs. Ref.: HR 6.99, 2.26–21.63), and elevated GGT (HR 2.14, 1.33–3.43). For ESRD due to OD we found an increased risk with male sex (HR 1.56, 1.13–2.15), increasing TG levels over 1.17 mmol/L and elevated TC (≥6.22mmol/L vs. Ref.: HR 1.56. 1.02–2.41).

**Fig 1:**
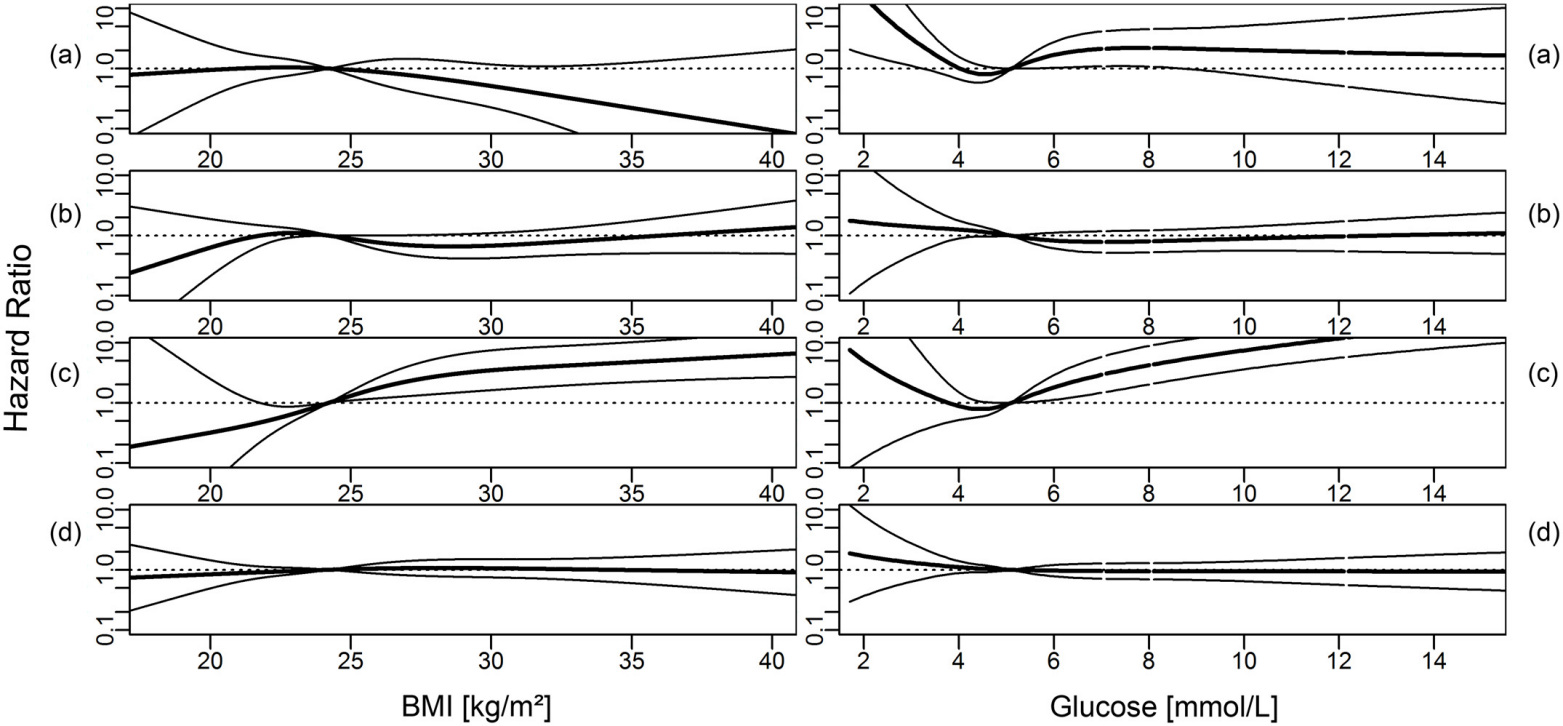
Hazard ratio (dark line, 95% CI: light lines) for cause-specific ESRD for body mass index (BMI) and blood glucose levels, modeled using cubic restricted splines. (a) ADPKD, (b) vascular nephropathy, (c) diabetic nephropathy and (d) other diseases. Medians of metabolic parameters were used as reference. Models were adjusted for age, sex, smoking status and mutually adjusted for metabolic parameters (as linear terms, logarithmized for triglycerides and gamma-GT).

**Fig 2:**
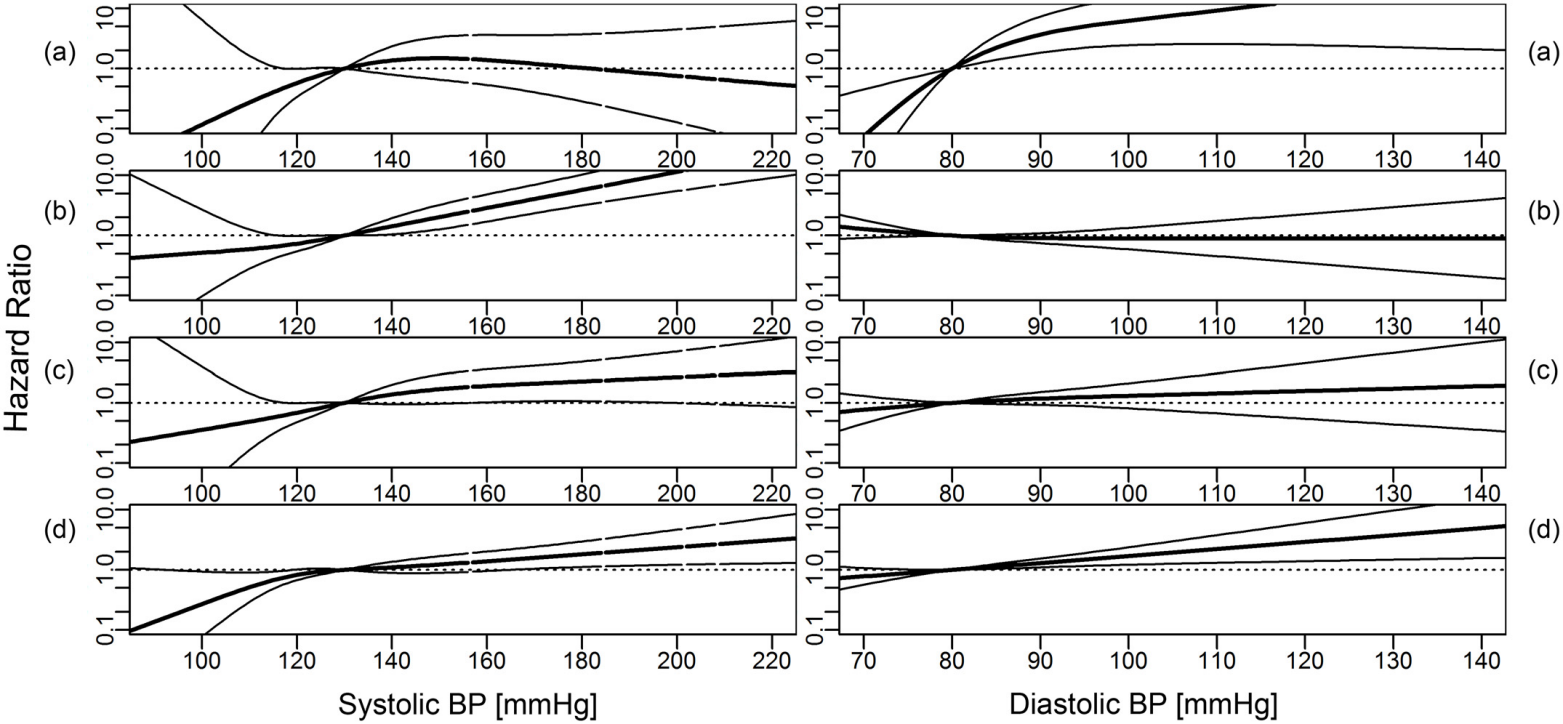
Hazard ratio (dark line, 95% CI: light lines) for cause-specific ESRD for systolic and diastolic blood pressure (BP), modeled using cubic restricted splines. (a) ADPKD, (b) vascular nephropathy, (c) diabetic nephropathy and (d) other diseases. Medians of metabolic parameters were used as reference. Models were adjusted for age, sex, smoking status and mutually adjusted for metabolic parameters (as linear terms, logarithmized for triglycerides and gamma-GT).

**Fig 3:**
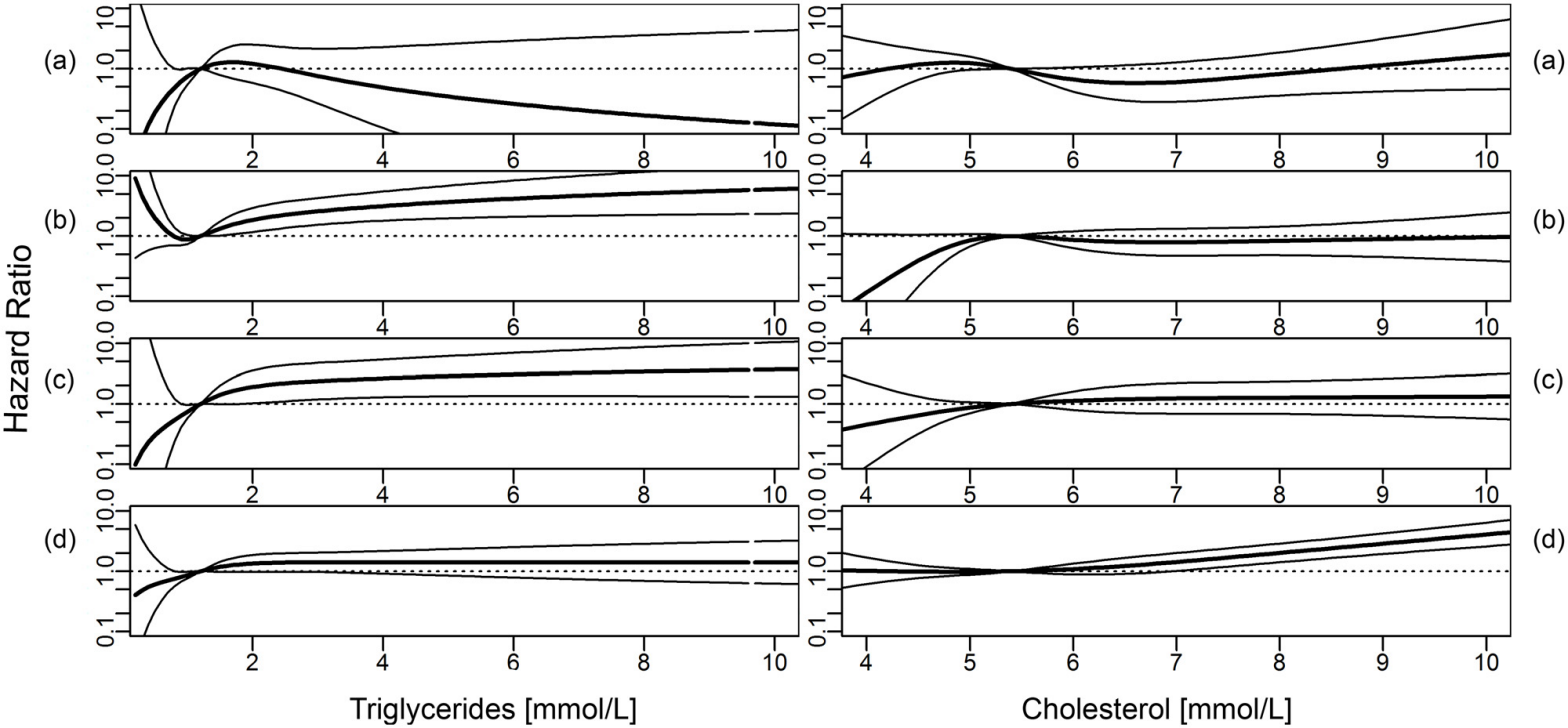
Hazard ratio (dark line, 95% CI: light lines) for cause-specific ESRD for triglycerides and total cholesterol levels, modeled using cubic restricted splines. (a) ADPKD, (b) vascular nephropathy, (c) diabetic nephropathy and (d) other diseases. Medians of metabolic parameters were used as reference. Models were adjusted for age, sex, smoking status and mutually adjusted for metabolic parameters (as linear terms, logarithmized for triglycerides and gamma-GT).

Overall, the analyses for nonlinear effects using cubic restricted splines confirmed the results of the Cox proportional hazards models for linear effects (Figs 1–3). An inverse association between BMI and ESRD due to ADPKD was found (Fig 1). The associations between increasing BMI, FBG and BPsys and increasing risk for ESRD by DN were found to be particularly strong (Fig 2). ESRD caused by VN was associated with increasing BPsys and increasing TG concentrations (Fig 3).

## Discussion

Against all earlier predictions, the number of incident end-stage renal disease (ESRD) patients in industrialized countries has stabilised or even decreased in recent years. In Austria, for example, the number of incident patients has dropped from 151 per million population (pmp) in 2007 to 143 pmp in 2013 [1]. This decline is attributed mostly to a reduction in the number of patients with DN. The annual incidence of these patients dropped from 50.2 in 2006 to 35.7 pmp in 2013 [17]. Similar trends are also reported from the United States, at least in Caucasian patients [2]. Over the last two decades the relative incidence of ESRD caused by DN (related to the total number of patients with diabetes) has declined by 28.3% in the United States [18]. Contrarily, the ESRD incidence rate in patients with other primary renal diseases such as ADPKD or glomerulonephritis remains fairly constant [1]. An analysis of the ERA-EDTA registry showed that from 1991 to 2010 the incidence rate of ESRD due to ADPKD increased slightly from 7.6 to 8.3 pmp and that the mean age at onset of RRT increased from 56.6 to 58 years. The authors concluded that currently there is no effective renoprotective treatment for ADPKD [19]. This discrepancy suggests that, depending on the primary renal disease, risk and progression factors are amenable to intervention. In renal diseases such as DN ESRD prevention approaches are likely to be more successful than in other diseases such as ADPKD, where current therapeutic interventions are largely ineffective.

We recently showed in this patient cohort that anthropometric and metabolic factors such as BMI, smoking, blood pressure, FBG, TG and TC are gender- and time-dependent risk factors for future all-cause ESRD [10]. The aim of the present investigation was to evaluate the association between these parameters and ESRD according to the primary renal disease. To the best of our knowledge, we are not aware of any previous population-based study that has addressed this question.

In our cohort male sex was associated with a 50% higher risk for ESRD, a phenomenon found in many other registries. The reason for male preponderance in ESRD programs is unclear and currently under discussion [20]. We found that male dominance is present in VN and OD, but not in ADPKD and DN. In a much larger database of the OEDTR including 6666 patients with DN, however, a 60% male predominance was clearly present in diabetes, too [17]. Data from the ERA-EDTA registry also suggest that in ADPKD on renal replacement therapy there is only marginal male dominance [19]. Of the whole ESRD population in that study, 61% were male, whereas among the ADPKD patients only 53% were male. In addition, the effect of sex on disease progression may differ between primary renal diseases. In general, a more rapid decline in GFR in men has been observed in diabetic and non-diabetic kidney disease [21–23]. In a meta-analysis on progression of non-diabetic kidney disease the lowest effect of sex if any has been described in ADPKD patients (Neugarten). Furthermore, the mean age of ADPKD patients entering renal replacement therapy differs by 1.1 years (57.5 years in men and 58.6 years in women) [19]. Therefore in ADPKD male sex does not seem to be associated with a more rapid decline in GFR.

Blood pressure has been identified as a frequent cause of chronic kidney disease (CKD) and a major CKD progression factor in epidemiologic studies [5, 8, 24–27]. We found a more than two-fold higher prevalence of hypertension in individuals who later developed ESRD. Overall, hypertension was associated with 161% higher risk for ESRD. Hypertension appeared to be a risk or progression factor in all disease groups, which underlines the importance of blood pressure control in all patients with CKD.

The effect of lipid levels on CKD progression has been generally reported to be negligible [28]. Surprisingly, we found that TG levels were a potent risk factor for all-cause ESRD and all cause-specific ESRD groups, whereas high TC was associated with higher risk only in the group of patients with OD. To our knowledge, there are no published data on TG levels as risk factor for ESRD. Reports from the Framingham Offspring Study, which showed TG to be associated with the development of CKD, and from the Atherosclerosis Risk in Communities Study, which described an association between TG and future decline in glomerular filtration rate (GFR) are in line with our findings [27, 29]. Clearly, this association requires confirmation in future studies. In addition, therapeutic reduction of TG levels may be a target for intervention that is worth being tested in a clinical trial.

Smoking is another well-established risk factor for ESRD. Hallan and Orth reported an adjusted HR for renal failure of 3.32 in former and 4.01 in current smokers in a Norwegian population study with a ten-year follow-up [9]. Recently, smoking has been reported to be associated with a two-fold higher risk for death from renal failure in a large U.S. cohort study [30]. Compared to these studies, the approximately 30% increase in ESRD risk in smokers found in our study is rather moderate. Smoking seems to be especially associated with VN and DN. The HR in ADPKD did not reach statistical significance, probably due to the small number of patients. In larger cohorts, however, smoking has been found to be a progression factor in ADPKD [31]. Smoking cessation clearly is of paramount importance in all patients at risk for or suffering from chronic kidney disease. Avoiding ESRD is a valuable treatment goal among others, such as risk reduction for vascular disease, pulmonary diseases and malignancy.

High body mass index or obesity is another parameter associated with future ESRD [3, 4, 7, 32, 33]. In our study, the baseline prevalence of obesity was 11% in all individuals, 21% in those who developed all-cause ESRD and 43% in patients with ESRD due to DN. However, multivariate analysis appeared to show the disease-specific effect to be limited to DN. Obesity, therefore, seems to be a risk factor for ESRD driven mainly by DN. These data are in line with a recent study that reported an association between higher BMI and ESRD only in individuals with metabolic syndrome [34].

The factors associated with ESRD due to DN in our study, such as elevated BMI, triglycerides, hyperglycemia, and hypertension, are modifiable targets for preventive and therapeutic interventions. Measures to improve these factors may eventually lead to a decrease in DN as a cause of ESRD, a phenomenon already being observed [1, 2, 18]. These measures include control of glycemia and blood pressure, particularly with blockers of the renin-angiotensin system [35–40]. Prescription of these drugs has increased dramatically in patients with type 2 diabetes over recent years [41]. Such interventions are also recommended by recent guidelines for the treatment of patients with diabetes [42, 43]. In particular, an intensified multifactorial intervention addressing all known risk factors for macrovascular and microvascular complications seems to be the most effective approach [44, 45].

Elevated GGT was found to be predictive of cardiovascular disease, the metabolic syndrome, type 2 diabetes, hypertension and chronic kidney disease [46]. Our results of an association between elevated GGT levels and ESRD related to diabetic kidney disease is consistent with previous findings [47, 48]. These studies found an association between GGT and development of albuminuria in high risk patients with diabetes or hypertension. The link between GGT and hypertension, diabetes and ESRD is speculative. High GGT is a marker of non-alcoholic fatty liver disease, which is strongly associated with the metabolic syndrome.

In ADPKD, to the contrary, these factors are not associated with the progressive decline in GFR [49]. Accordingly, the measures taken to halt progression in other renal diseases do not seem to be very effective in ADPKD, and hence the renal prognosis of these patients has not changed over the last decades [19, 50, 51]. Of course, ADPKD is a hereditary disease and the parameters in our study are relevant only for progression, but not initiation of CKD. The most important factor certainly is the disease genotype. In patients with a PKD2 mutation renal survival is around twenty years longer than in those with a PKD1 mutation [52]. The role of blood pressure in ADPKD is still debated. A recent review describes early-onset and severity of hypertension as being associated with progression to ESRD, but high blood pressure may be only a marker of a more severe genotype [49]. Moreover, in the HALT-PKD trial rigorous blood pressure control, as compared to standard care, was associated with a slower increase in total kidney volume, but had no effect on the decline in estimated GFR [50].

In our study, vascular nephropathy had a risk profile comparable to DN, with smoking, hypertension and elevated triglycerides as associated risk factors. It would appear that targeted interventions can reduce the incidence of VN. However, in the Austrian Dialysis and Transplant Registry the proportion of VN among incident ESRD patients has increased from 20% in 2006 to 26% in 2013, which contrasts the trend in DN [1]. We are unaware of studies on the natural course of VN or randomized controlled trials of interventions to halt progression in VN. The CORAL trial examined the effect of stenting a severe atherosclerotic renal artery stenosis as compared to medical therapy alone [53]. Medical therapy consisted of antihypertensive treatment with candesartan with or without hydrochlorothiazide, and amlodipine with a blood pressure goal of <130/80 mmHg in CKD patients, and atorvastatin. Despite this antihypertensive and cholesterol-lowering treatment with or without stenting, around 15% of patients experienced a loss of eGFR of more than 30% over a median of 43 months. These data suggest that prevention of progression may be more difficult to achieve in VN than in DN. In addition, ESRD due to VN is a disease of older age (mean age at ESRD in our study was 71 years in VN as compared to 66 years in DN).

Our data in the group of other diseases (mainly glomerulonephritis, interstitial nephritis and other hereditary diseases) suggest that despite another underlying disease mechanism such as inflammation or a hereditary defect, progression factors such as hypertension and hyperlipidemia are of importance and represent critical treatment targets. At least blood pressure control using ACE inhibitors is highly effective in glomerulonephritis and Alport syndrome [54, 55].

Major strengths of our study are its large sample size and the long follow-up time, which allowed us to analyze renal disease subgroups. However, the number of cases in the individual groups, especially in the ADPKD group, is rather small. One major limitation of our study is the lack of renal function parameters such as serum creatinine, or markers of renal injury such as albuminuria. Unfortunately, these tests were not included in the VHM&PP health examinations We therefore do not know whether probands already had chronic kidney disease at baseline examination and inclusion in the study. Restriction of the data to measurements after 1988 did not substantially change the results.

In summary, we found that in a large population-based cohort risk factors for ESRD are to some extent disease-specific. These differences may explain why current measures to prevent or slow progression to ESRD are successful for some diseases such as diabetic nephropathy, but disappointingly ineffective for others such as ADPKD.

## Abbreviations

ESRD: end-stage renal disease
VIVIT: Vorarlberg Institute for Vascular Investigation and Treatment
VHM&PP: Vorarlberg Health Monitoring & Prevention Programme
BMI: body mass index
FBG: fasting blood glucose
TC: total cholesterol
TG: triglyceride
GGT: gamma-glutamyl transferase
HR: hazard ratio
ADPKD: autosomal dominant polycystic kidney disease
VN: vascular/hypertensive nephropathy
DN: diabetic nephropathy
OD: other diseases
RRT: renal replacement therapy
HE: health examination
BPsys: systolic blood pressure
BPdia: diastolic blood pressure
BG: blood glucose
OEDTR: Austrian Dialysis and Transplant Registry
ERA-EDTA: European Renal Association-European Dialysis and Transplant Association
CI: confidence interval
Ref.: reference
pmp: per million population
CKD: chronic kidney disease
GFR: glomerular filtration rate
PKD1: Polycystic Kidney Disease 1
PKD2: Polycystic Kidney Disease 2
HALT-PKD: Halt Progression in Polycystic Kidney Disease Trial
ACE: angiotension converting enzyme
